# YKL-40 acts as an angiogenic factor to promote tumor angiogenesis

**DOI:** 10.3389/fphys.2013.00122

**Published:** 2013-05-28

**Authors:** Rong Shao

**Affiliations:** ^1^Molecular and Cellular Biology Program, Morrill Science Center, University of MassachusettsAmherst, MA, USA; ^2^Department of Veterinary and Animal Sciences, University of MassachusettsAmherst, MA, USA

**Keywords:** YKL-40, angiogenesis, VEGF, tumor cells, vascular endothelial cells, tumor-associated macrophages, tumor microenvironment, neutralizing anti-YKL-40 antibody

## Abstract

A secreted glycoprotein YKL-40 also named chitinase-3-like-1 is normally expressed by multiple cell types such as macrophages, chondrocytes, and vascular smooth muscle cells. However, a prominently high level of YKL-40 was found in a wide spectrum of human diseases including cancers and chronic inflammatory diseases where it was strongly expressed by cancerous cells and infiltrating macrophages. Here, we summarized recent important findings of YKL-40 derived from cancerous cells and smooth muscle cells during tumor angiogenesis and development. YKL-40 is a potent angiogenic factor capable of stimulating tumor vascularization mediated by endothelial cells and maintaining vascular integrity supported by smooth muscle cells. In addition, YKL-40 induces FAK-MAPK signaling and up-regulates VEGF receptor 2 in endothelial cells; but a neutralizing antibody (mAY) against YKL-40 inhibits its angiogenic activity. While YKL-40 is essential for angiogenesis, little is known about its functional role in tumor-associated macrophage (TAM)-mediated tumor development. Therefore, significant efforts are urgently needed to identify pathophysiological function of YKL-40 in the dynamic interaction between tumor cells and TAMs in the tumor microenvironment, which may offer substantial mechanistic insights into tumor angiogenesis and metastasis, and also point to a therapeutic target for treatment of cancers and other diseases.

## Introduction

YKL-40 is a 40-kDa secreted glycoprotein discovered as a heparin-binding protein and belongs to the chitinase gene family that binds to chitin-like oligosaccharides (Shackelton et al., 1995; Hu et al., 1996; Fusetti et al., 2003). However, it does not have chitinase/hydrolase activity because of the substitution of an essential glutamic acid with leucine in the chitinase-3-like catalytic domain (Renkema et al., 1998; Fusetti et al., 2003). YKL-40 is normally expressed by a number of different cell types including chondrocytes (Hu et al., 1996), synoviocytes (Nyirkos and Golds, 1990), vascular smooth muscle cells (Shackelton et al., 1995), macrophages (Rehli et al., 1997), and neutrophils (Kzhyshkowska et al., 2007), and it has been recognized as a growth factor capable of stimulating connective tissue cell growth and endothelial cell migration, and inhibiting mammary epithelial cell differentiation (Malinda et al., 1999; De Ceuninck et al., 2001; Recklies et al., 2002; Scully et al., 2011). However, the pathophysiological function of YKL-40 is still not fully understood.

Growing evidence has indicated that expression levels of YKL-40 are elevated in multiple human diseases including type 2 diabetes (Persson et al., 2012), obesity and insulin resistance in children (Kyrgios et al., 2012), Alzheimers' diseases (Perrin et al., 2011), heart failure (Harutyunyan et al., 2012), and other cardiovascular disorders (Kjaergaard et al., 2010). In addition, elevated YKL-40 was found in a vast array of inflammatory diseases that contain bacterial infections (Kronborg et al., 2002), rheumatoid arthritis (Nielsen et al., 2011), osteoarthritis (Volck et al., 2001), hepatic fibrosis (Pizano-Martinez et al., 2011), and hepatitis (Johansen et al., 2000; Fontana et al., 2010), asthma and chronic obstructive pulmonary diseases (Park et al., 2010), neuroinflammation (Bonneh-Barkay et al., 2010), and bowel lesion (Vind et al., 2003). In the chronic inflammatory diseases, YKL-40 is appreciated to mediate infiltration, differentiation, and maturation of macrophages, the primary leukocytes in response to inflammation (Boot et al., 1995; Rehli et al., 1997; Renkema et al., 1998; Rehli et al., 2003). The cytokines colony-stimulating factor-1 and granulocyte macrophage colony-stimulating factor, essential for macrophage recruitment, displayed the ability to induce 180–200 fold higher levels of YKL-40 mRNA transcripts in macrophages, thus rendering infiltrating macrophages mature (Hashimoto et al., 1999; Suzuki et al., 2000). Studies with YKL-40 deficient mice offered strong evidence supporting the role of YKL-40 in macrophage activity, as these mice exhibited markedly diminished antigen-induced Th2 inflammation and impaired macrophage activation and differentiation (Lee et al., 2009).

**Figure 1:**
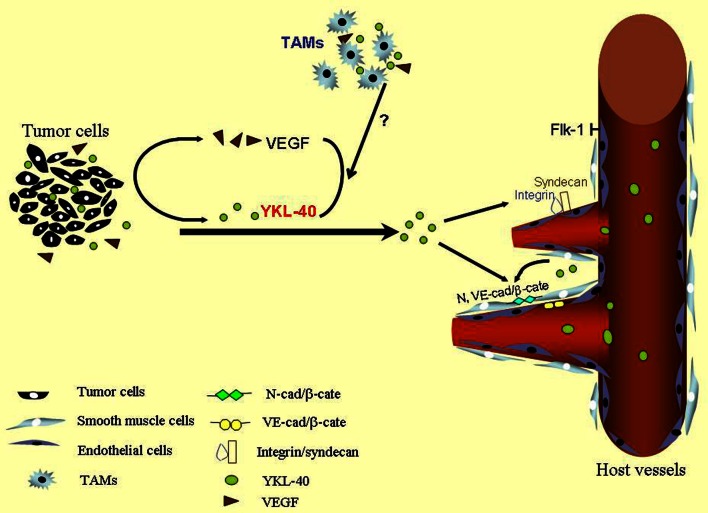
**A scheme for YKL-40-induced tumor angiogenesis.** YKL-40 secreted from tumor cells stimulates vascular endothelial cell activation to induce tumor angiogenesis through membrane receptor coupling of syndecan-1 with integrin. YKL-40 regulates VEGF in tumor cells and both may synergistically promote endothelial cell angiogenesis. YKL-40 derived from smooth muscle cells also controls vessel stability and permeability via inducing association of N- and VE-cad with β-catenin (β-cate) expressed by smooth muscle cells and endothelial cells, respectively. TAMs participate in the vascular development probably through YKL-40, which warrants further investigation. Green balls indicate secreted YKL-40 and brown triangles represent VEGF.

Over the past decades, multiple independent studies have demonstrated that high serum levels of YKL-40 are correlated with metastasis and poor survival in a variety of human carcinomas such as breast cancer (Jensen et al., 2003), colorectal cancer (Cintin et al., 1999), ovarian cancer (Hogdall et al., 2003), leukemia (Bergmann et al., 2005), lymphoma (Hottinger et al., 2011), and glioblastoma (GBM) (Pelloski et al., 2005), suggesting that serum levels of YKL-40 serve as a diagnostic and prognostic cancer biomarker. YKL-40 is expressed by both tumor cells and their surrounding tumor infiltrating macrophages also named tumor-associated macrophages (TAM) that produce various tumor-promoting factors including angiogenic factors [vascular endothelial growth factor (VEGF), epidermal growth factor (EGF), basic fibroblastic growth factor (bFGF), platelet-derived growth factor (PDGF)] (Chong et al., 1999; Ganapathy et al., 2010), cytokines (IL-1, IL-6) (Wang et al., 2009; Pini et al., 2012), and chemokines (CCL-2, CCL-18, CXCL-12) (Dubinett et al., 2010; Chen et al., 2011a,b,c; Fridlender et al., 2011; Boimel et al., 2012). Although the overall pathological role and molecular mechanisms of YKL-40 in tumorigenesis remain to be established, an angiogenic feature has been reported to regulate tumor development in breast cancer, colon cancer, and GBM (Shao et al., 2009; Francescone et al., 2011; Kawada et al., 2012). Here, this review primarily focused on the angiogenic signature of YKL-40 derived from tumor cells and smooth muscle cells, as a model is illustrated in Figure 1, while a potential distinct role of YKL-40 in TAM-mediated tumor development warrants further investigation.

## An angiogenic signature of YKL-40

Due to lack of its chitinase activity, the pathological role of YKL-40 in cancer development has not been substantially explored yet. Gp38k, a YKL-40 homolog, was found to induce endothelial cell migration, indicative of angiogenic activity (Nishikawa and Millis, 2003). To evaluate if YKL-40 possesses the same angiogenic activity in cancer, a breast cancer line MDA-MB-231 and colon cancer lines HCT-116 and SW480 were engineered to express ectopic YKL-40 (Shao et al., 2009; Kawada et al., 2012). Xenotransplantation of YKL-40-expressing tumor cells gave rise to 4–8 fold larger tumors than ones formed from their corresponding control cells, while acquired expression of YKL-40 did not predispose these cells toward increased proliferation in the cultured condition. Immunohistochemical studies indicated that levels of blood vasculature formed in YKL-40-expressing MDA-MB-231, HCT-116, and SW480 tumors were 1.8–2.0 fold greater than those in control tumors, suggesting that YKL-40 acts as an angiogenic factor to promote vessel formation and tumor growth. Such an angiogenic capability of YKL-40 was also validated in GBM (Francescone et al., 2011), the most lethal primary brain tumor characterized by vigorous vascularization (Wen and Kesari, 2008). For example, YKL-40-directed gene knockdown in GBM-derived U87 cells notably suppressed tumor angiogenesis, as the vessel density of YKL-40 shRNA tumors was decreased to 44% of vasculature relative to control tumors and tumor volume was accordingly reduced to approximately 30% of control counterparts (Shao et al., 2009). All of these multiple *in vivo* approaches demonstrate the angiogenic signature of YKL-40 in the tumor development, based on these xenografts carrying different levels of YKL-40. However, this angiogenic phenotype may also involve tumor-promoting function of host-derived cells in the tumor microenvironment, as increased infiltrating macrophages were observed in the YKL-40-expressing tumors, but not in the control tumors (Kawada et al., 2012). It will be interesting to know if these macrophages also increase to produce YKL-40 that enhances the angiogenesis induced by tumor-derived YKL-40.

To monitor its direct effects on vascular endothelial cells, conditioned media derived from both MDA-MB-231 and HCT-116 cells ectopically expressing YKL-40 or vector were introduced to human microvascular endothelial cells (HMVEC) and tested for endothelial cell angiogenic activity *in vitro*. Analogous to the findings in animals, both YKL-40-producing tumor cells induced endothelial cell migration and tube formation (Shao et al., 2009). Likewise, SW480 over-expressing YKL-40 also enhanced migration and tube formation of human umbilical vein endothelial cells by 1.4–2 fold greater than the control cells expressing vector (Kawada et al., 2012). YKL-40 gene knockdown abrogated these angiogenic activities. In addition, conditioned medium of U87 cells expressing YKL-40 shRNA inhibited the angiogenic activities of HMVEC vs. control cell medium (Francescone et al., 2011). To further support these *in vitro* data and firmly establish the angiogenic signature for YKL-40, recombinant YKL-40 was created and characterized for the angiogenic activity. YKL-40 stimulated endothelial cell migration and tube formation approximately 3–4 fold greater than control cells, the angiogenic capability identical to VEGF, one of the most potent angiogenic factors (Shao et al., 2009). It was noted that most of these cultured concentrations of YKL-40 between 100 and 200 ng/ml were based on serum levels of YKL-40 observed in cancer patients (Jensen et al., 2003; Johansen et al., 2003). However, it is unclear if these concentrations indeed reflect YKL-40 levels in the local tumor, because the serum levels are probably derived from multiple organs and also involve the dilution effect. Therefore, a cautious interpretation from these cultured systems should be considered in stimulating YKL-40's action *in vivo*. Nevertheless, all these animal and cultured data suggest that YKL-40 acts as an angiogenic factor to trigger tumor vascular development.

## Relationship between YKL-40 and VEGF

In the tumor microenvironment, a significant amount of angiogenic factors are secreted from tumor cells and activate adjacent vascular endothelial cells to induce angiogenic responses by means of a paracrine loop (Hanahan and Weinberg, 2010). YKL-40 and VEGF are believed to be mainly derived from tumor cells and both display strong angiogenic activities in tumor development, but their regulatory relationship has not been revealed until recently. YKL-40-induced endothelial cell angiogenic responses in culture were VEGF-independent, as an anti-VEGF neutralizing antibody failed to impede YKL-40-induced migration and tube formation of HMVECs (Shao et al., 2009). This data suggests that YKL-40 and VEGF individually promote endothelial cell angiogenesis. U87 brain tumor cells were found to express high levels of YKL-40 and VEGF (Francescone et al., 2011). When YKL-40 expression was inhibited via small hairpin RNA (shRNA), a reduction of VEGF was subsequently obtained in these tumor cells, indicative of a regulatory role of YKL-40 in VEGF production. In light of a potential similar role of VEGF in YKL-40 expression, transient neutralization of VEGF using a neutralizing anti-VEGF antibody for 24 h did not have impacts in YKL-40 production. Interestingly, inhibition of VEGF for 1 week noticeably induced expression of YKL-40, the unexpected event identical to the documented evidence using VEGF shRNA in U87 cells (Saidi et al., 2008). These results imply that VEGF does not regulate YKL-40, but a long-term blockade of VEGF may result in angiogenic compensative activities of tumor cells by inducing YKL-40. It is most likely that a long course of the stress caused by blockade of one growth factor and/or angiogenic factor commits the cells to induce expression of other potent angiogenic factors in order for cell survival and function. It was noted that these tumor cells such as brain tumor cells express a high level of angiogenic factors able to promote vascular development (Junker et al., 2005a,b; Francescone et al., 2011). This phenomenon was identically observed in a number of tumor models treated chronically with a single anti-angiogenic drug, the event known as angiogenic rebound (see below). However, it needs to determine if this angiogenic switch is unique for highly angiogenic tumors, but not for other non-angiogenic tumors.

Apart from their relationship defined earlier in cultured cancer cell lines, studies on human cancers also suggest the similar association of YKL-40 with VEGF in tumor angiogenesis. Tumor specimens from 12 cases of patients with GBM were used to test the relationship between YKL-40 and VEGF (Francescone et al., 2011). Expression of YKL-40 and VEGF in tumor samples displayed a trend toward positive correlation (*p* = 0.062), but a larger sample pool sufficient to establish their relationship is required. In context with the findings *in vitro*, all the evidence suggests that YKL-40 regulates VEGF in tumor cells and both may exert a synergistic impact in tumor vascularization (Figure 1).

A chronic course of angiogenic blockade in either YKL-40 or VEGF may not receive a full elimination of tumor angiogenesis; instead, an unexpected compensation by the other factor may lead to an opposite outcome including resistance to the single-factor treatment and angiogenic rebound. Indeed, the theme of this anti-angiogenic bypass upon a chronic single treatment has been supported by a number of strong evidence documented in pre-clinical and clinical trials. For instance, individual anti-angiogenic treatment with bevacizumab (anti-VEGF antibody, Avastin), DC101 (anti-VEGF receptor antibody), or sunitinib (anti-VEGF receptor kinase inhibitor) can elicit vascular rebound and tumor cell invasiveness and metastasis in several animal models (Casanovas et al., 2005; Ebos et al., 2009; Paez-Ribes et al., 2009). In clinical trials, the benefit of anti-angiogenic agents (e.g., sunitinib, bevacizumab) appears to be transitory in the treatment of several types of advanced cancers, as drug resistance, tumor regrowth, and extensive vascular recovery rapidly develop, once the therapy is terminated (Bergers and Hanahan, 2008; Burstein et al., 2008; Verhoeff et al., 2009; Wick et al., 2010). In addition, it is noteworthy that bevacizumab has been removed by the Food and Drug Administration from monotreatment of metastatic breast cancers, based on insufficient amelioration of patient overall survival. While it is emerging that a monotherapy against a single factor could unexpectedly result in conflicting outcomes, it is still enigmatic if YKL-40 acts as a major factor to contribute to the angiogenic rebound in these patients that are treated with one drug such as bevacizumab. Nevertheless, to prevent either anti-VEGF or possible anti-YKL-40 resistance, it should be taken into account for a combined regimen with anti-VEGF and anti-YKL-40 therapies in cancer patients.

## Molecular mechanisms of YKL-40 in endothelial cells and tumor cells

**Figure 2:**
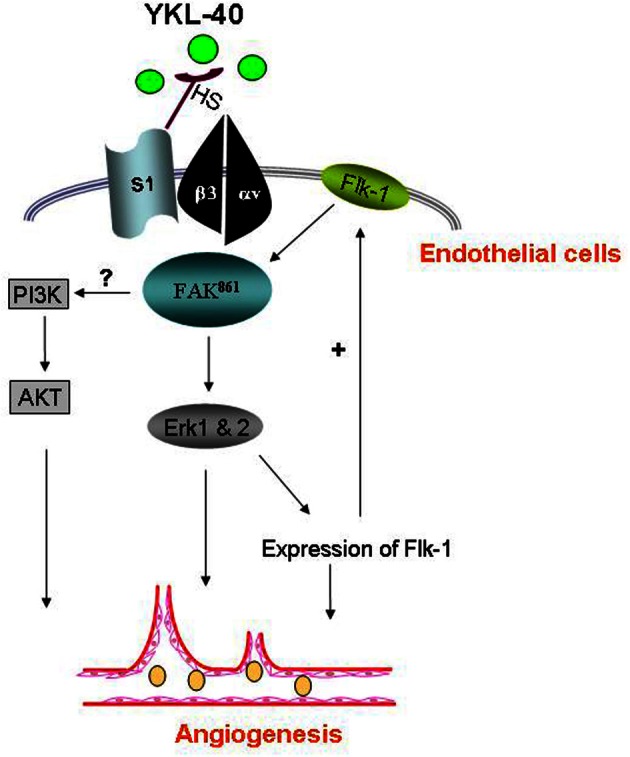
**YKL-40 induces angiogenic signaling in endothelial cells.** YKL-40 induces the coordination of syndecan-1 (S1) and integrin α_v_β_3_ through binding heparan sulfate chains (HS) of S1 on cell surface. The intracellular signaling pathway includes pFAK^861^ and downstream MAP kinase Erk 1 and 2, leading to angiogenic responses and angiogenic gene expression as well (e.g., Flk-1). Flk-1 up-regulation in turns activates the signal transduction cascade, constituting a positive feedback loop to enhance angiogenic responses. Elevated Flk-1 may also sensitize angiogenic responses to VEGF. An additional PI3K-AKT pathway participating in YKL-40-induced angiogenesis in endothelial cells warrants further investigation.

Although membrane receptors specific for YKL-40 binding remain to be identified, heparin-binding affinity of YKL-40 seems to be essential for its activity, resembling the heparin-binding property of other secreted proteins such as extracellular matrix protein vitronectin and angiogenic factors bFGF and VEGF (Bernfield et al., 1999; Beauvais et al., 2004). The heparin binding affinity is at least approximately 100-fold lower (disassociation constant Kd ~10^−8^ – 10^−9^ M) than their specific receptor binding (Kd ~10^−11^ – 10^−12^ M), but this binding can facilitate their adjacent specific receptor binding (Baird et al., 1988; Park et al., 2000; Prince et al., 2010). Syndecan-1, a transmembrane receptor, is the major source of cell surface heparan sulfate (HS). There is compelling evidence demonstrating that endowed with the HS chain on its ectodomains, syndecan-1 acts as a matrix co-receptor with adjacent membrane-bound receptors such as integrins to mediate cell adhesion and/or spreading (McQuade et al., 2006). This co-membrane receptor model of syndecan-1 with integrin was found to play an indispensable role in mediating YKL-40-induced angiogenic responses (Shao et al., 2009). YKL-40 can induce coupling of syndecan-1 with integrin α_v_β_3_ through binding to HS and then activate intracellular signaling effectors focal adhesion kinase (FAK^861^) and mitogen-activated protein kinase (MAPK) that regulate endothelial cell adhesion and motility (Figure 2). In addition, treatment of HMVEC with recombinant YKL-40 increases protein expression and active form of both VEGF receptor 2 (Flk-1) and intracellular extracellular signal-regulated kinase (Erk 1 and 2) that in turn enhance angiogenic signaling pathways (Faibish et al., 2011; Lee et al., 2011). Furthermore, an additional phosphoinositide 3-kinase-protein kinase B (PI3K-AKT) pathway responsible for YKL-40's action in vascular endothelial cells is proposed, but no date has confirmed it yet.

The signaling activation by YKL-40 in endothelial cells was similarly identified in the tumor line U87 cells, in which YKL-40 induces strong association of syndecan-1 with different integrin α_v_β_5_ and downstream activation of FAK^397^ and Erk 1 and 2, thus targeting VEGF expression that evokes endothelial cell angiogenesis (Francescone et al., 2011). In apoptotic responses, YKL-40 was found to prevent U87 cell death from γ-irradiation through activation of PI3K-AKT pathways, the signal transduction identical to the cascade that mediates YKL-40-induced mitogenesis in connective tissue cells (Recklies et al., 2002). In SW480 cells, YKL-40 also regulated MAPK including Erk 1, 2, and JNK that induce expression of IL-8 and monocyte chemoattractant protein-1, facilitating angiogenesis (Kawada et al., 2012). Therefore, YKL-40 acts as an angiogenic factor and a growth factor to induce distinct signaling cascades in endothelial cell angiogenesis and tumor cell survival, respectively.

## Activity of YKL-40 in vascular smooth muscle cells

As discussed earlier, neo-vascular development is mainly ascribed to the activation of vascular endothelial cells, the primary component of blood vessels. However, a functional role of YKL-40 in smooth muscle cells or vascular pericytes, another subset of vascular cell populations that support vessel integrity and stability, is poorly understood, even those cells express YKL-40. Using a smooth muscle cell model named glioblastoma stem-differentiated cells (GSDC), we found that YKL-40 enhances both GSDC and HMVEC contacts, restricts vascular leakage, and stabilizes vascular networks (Francescone et al., 2013). Furthermore, the vascular sprouting and stability mediated by smooth muscle-like cells are dependent on signaling activation induced by YKL-40, which includes interaction of membrane adhesion molecules neural cadherin (N-cad) with β-catenin and downstream intracellular cytoskeleton smooth muscle alpha actin (Figure 1). Likewise, adhesion and permeability of HMVECs regulated by YKL-40 rely on the interaction of vascular endothelial cadherin (VE-cad) with β-catenin and downstream effector actin. YKL-40 gene knockdown in GSDCs leads to disruption of association of VE-cad with β-catenin and increases endothelial cell permeability via a paracrine manner. In GSDCs, YKL-40 shRNA also inhibits interaction of N-cad with β-catenin and reduces GSDC-mediated vessel stability, suggesting that both vascular cell populations regulated by YKL-40 coordinately contribute to the angiogenesis. Furthermore, xenotransplantation of GSDCs expressing YKL-40 shRNA in mice gives rise to impaired blood vessel integrity with collapsed vessel lumens and diminished smooth muscle-like cell coverage; whereas control GSDCs develop extensive and stable blood vessels covered with more smooth muscle-like cells, highlighting a unique role of YKL-40 derived from smooth muscle-like cells in the maintenance of vascular permeability, stability, and angiogenesis. Although the interaction between cadherins and catenin is vital for YKL-40's function, it is still unknown whether or not this interaction is dependent on pre-activation of syndecan-1 that is for YKL-40 binding on the membrane.

## Functional blockade of YKL-40—a potential tool for anti-angiogenic therapy

A neutralizing anti-YKL-40 antibody (named mAY) from mice immunized against recombinant YKL-40 was recently established (Faibish et al., 2011). HMVEC migration and tube formation induced by YKL-40 in a dose-dependent fashion were markedly suppressed by mAY. mAY was also found to abolish YKL-40-induced activation of Flk-1 and intracellular signaling MAP kinase Erk 1 and Erk 2 in HMVEC. In addition, mAY facilitated death responses of the U87 glioblastoma cell line to γ-irradiation through decreased expression of pAKT and AKT (Faibish et al., 2011). Consistent with these data from cultured cells, tumor angiogenesis developed from xenografted U87 cells expressing YKL-40 was abrogated in mice treated with mAY, whereas vigorous angiogenesis was observed in mIgG-treated control tumors. Similar studies focusing on YKL-40 neutralization in the angiogenesis of colon cancer unveiled the identical importance for the anti-YKL-40 antibody (Kawada et al., 2012). Therefore, the evidence from such pre-clinical trials has hold therapeutic promise for formulating a humanized anti-YKL-40 antibody in the treatment of cancer patients as well as other possible diseases.

Chitin can bind to both chitinases that have hydrolase activity and chitinase-like proteins that lack the enzymatic activity such as YKL-40 (Lee et al., 2008). Size difference of chitin exhibits distinct capabilities of inducing host immune responses, as small particles (<10 μm) can induce TH1 type immune responses whereas large ones (>50 μm) activate TH2 type responses (Shibata et al., 1997; Lee et al., 2008). Recently, Iragavarapu-Charyulu's group has utilized small chitin to test a hypothesis that saturation of YKL-40's binding can alleviate its direct tumor-promoting effects on tumors (Libreros et al., 2012). Chitin (1–10 μm) has strong binding affinity with YKL-40 and is associated with activation of M1 type macrophages. This binding between chitin and YKL-40 may induce immune response shift from pro-tumorigenic TH2 type (M2 macrophage activation) to anti-tumorigenic TH1 type (M1 macrophage activation). They found that the treatment of mammary tumor-bearing mice with chitin significantly decreased serum levels and splenic macrophages of YKL-40, CXCL2, and MMP-9, thereof impeding lung metastasis. It remains to be determined if the reduction of YKL-40 expression and subsequent inhibition of tumor progression are different from treatment with large chitin. Other alternative possible approaches that block YKL-40 signaling pathways may also suffice to prevent YKL-40 activity or be synergistic in conjunction therapies with YKL-40-directed inhibitors. Nonetheless, the recent multiple animal approaches to blocking YKL-40 function offer therapeutic value potential for modalities of clinical patients.

## YKL-40 in human tumor angiogenesis

A multitude of clinical studies have revealed that serum levels of YKL-40 were elevated in patients with a series of carcinomas including breast (Jensen et al., 2003), colorectum (Cintin et al., 1999), ovary (Hogdall et al., 2003), prostate (Kucur et al., 2008), brain (Pelloski et al., 2005), and blood (Bergmann et al., 2005). These increased levels were correlated with poorer survival of cancer patients (Cintin et al., 1999, 2002; Hogdall et al., 2003; Jensen et al., 2003; Johansen et al., 2003; Bergmann et al., 2005; Pelloski et al., 2005), suggesting that serum levels of YKL-40 serve as a prognostic cancer biomarker (Johansen et al., 2009).

While amounting evidence was documented in the study of serum levels of YKL-40, there is relatively limited evidence focusing on YKL-40 expression in cancers, particularly for its association with angiogenesis. Thirty-eight cases of breast infiltrating ductal carcinomas were surveyed for relationship of YKL-40 with vessel formation using immunohistochemistry of CD34, a vascular endothelial cell marker (Shao et al., 2009). Of those 38 cancers, 23.7% (9 cases) contained high expression levels of YKL-40 and 23.7% (9 cases) displayed medium levels of YKL-40; whereas 52.6% (20 cases) were negative or low. These three groups with different expression levels of YKL-40 were found to be significantly correlated with different degrees of vascularization with CD34-positive vessels in tumor sections (*p* = 0.006), in which the blood vessel density of the two groups that demonstrated high and medium levels of YKL-40 were 2.1 and 1.6-fold greater than the group expressing low YKL-40, respectively. Consistent with this finding, of 61 colorectal cancer samples, 37 and 24 cases expressing strong YKL-40 and weak YKL-40 exhibited 2.0 and 1.6-fold higher microvessel density than did 12 normal subjects, respectively (Kawada et al., 2012). In addition, studying 11 cases of patients with GBM revealed that the higher the YKL-40 expression, the more extensive the vessels appeared to be (Francescone et al., 2011). All of the evidence demonstrates that YKL-40 expression in cancer is associated with vascular network development, underscoring the angiogenic property of YKL-40 identified in pre-clinical (cultured cells and xenografted animal models) and clinical studies.

In the study of YKL-40 expression and clinical outcomes, several independent studies with large breast cancer cohorts from different laboratories including ours demonstrate that YKL-40 expressed by breast cancer is associated with estrogen receptor (ER^−^), progesterone receptor (PR^−^), and human epidermal growth factor receptor 2 (Her2/*neu*) (Kim et al., 2007; Roslind et al., 2007b; Shao et al., 2011). Unexpectedly, cancer tissue expression, contrary to its levels in the blood, was not correlated with patient overall survival or disease-free survival in 8-year follow-up studies (Shao et al., 2011). This finding was reinforced by the others surveying 630 breast cancer patients (Roslind et al., 2007b). Interestingly, strong expression levels of YKL-40 were identified in TAMs in both breast cancer and lung cancer, as these TAMs surrounding tumor cells co-expressed YKL-40 and CD68, a marker of macrophages (Junker et al., 2005a,b; Roslind et al., 2007a; Stearman et al., 2008). It is well established that infiltrating macrophages play an essential role for angiogenesis in both inflammatory diseases and tumor development, because increased infiltration of macrophages leads to accumulation of multiple growth factors (TGF-β, EGF, bFGF, VEGF, and PDGF) that modulate tissue repair and angiogenesis (Chong et al., 1999; Ganapathy et al., 2010). Furthermore, increased macrophage density in cancers correlates with tumor angiogenesis and poorer patient survival (Leek et al., 1996, 2000; Bingle et al., 2002; Tsutsui et al., 2005). However, it remains to be clarified if the expression of YKL-40 by TAMs is associated with cancer metastasis and patient survival. Validating their relationship may provide a key role of TAMs in the contribution to cancer malignancy.

## Unanswered questions

It has been established that chronic inflammation is a key component of cancer development and metastasis (Coussens and Werb, 2002). TAMs, the primary infiltrating leukocytes, act as a core mediator to regulate inflammatory responses that exacerbate the pathogenesis of cancers (Coussens and Werb, 2002; Pollard, 2004; Lewis and Pollard, 2006). Although tumor-derived YKL-40 was reported to be associated with macrophage recruitment and angiogenesis in colorectal cancer (Kawada et al., 2012), we still lack sufficient knowledge regarding the functional role and molecular mechanisms of YKL-40 in TAM-mediated tumorigenesis. TAMs have the ability to render tumor cells invasive through up-regulation of multiple inflammatory factors such as cytokines, growth factors, chemokines, and metalloproteinases (MMPs). It is noted that YKL-40 is essential for macrophage differentiation and maturation (Rehli et al., 1997, 2003). Thus, it is intriguing to interrogate if the inflammatory responses mediated by these factors in the tumor microenvironment are dependent on TAM-derived YKL-40. For example, little is known if YKL-40 up-regulates inflammatory cytokines in TAMs, even though YKL-40 is recognized as an inflammatory factor and can induce IL-8 from tumor cells (Rathcke and Vestergaard, 2006; Qin et al., 2007; Kawada et al., 2012). YKL-40 can induce VEGF in tumor cells as discussed earlier, but the similar relationship in TAMs remains to be established. In addition, it is still unclear regarding the molecular mechanisms by which YKL-40 regulates macrophage recruitment, differentiation, and maturation. Identification of potential signaling mediators in TAMs may provide alternative approaches to block activities of TAMs, thus impeding tumor progression. Besides TAMs, other YKL-40-producing cells surrounding tumor cells and TAMs (e.g., neutrophils) should be not neglected in the tumor microenvironment, as these cell populations likely coordinate with tumor cells, TAMs, and vessel cells to facilitate tumor cell ectopic dissemination. The function of cell-associated YKL-40 in tumor may be different from free form of YKL-40 in the blood, because we currently do not know receptors or ligands for YKL-40 binding. This may also explain the difference between serum levels and cancer cell levels of YKL-40 in association with tumor malignancy. Thus, characterization of their relationship will aid in establishing a new therapeutic target for treatment.

YKL-40 harbors chitinase-3-like catalytic domains, but does not possess chitinase activities. Therefore, its functional domain(s) are still unclear. Chen at al., recently reported that a chitin-binding motif located between 325 and 339 amino acid residues at the C terminus of YKL-40 is critical for YKL-40 inflammatory activities including AKT-mediated cytokine production (IL-8 and TNF-α) in colonic epithelial cells (Chen et al., 2011a,b). This motif may be also vital for other activities of YKL-40 such as angiogenic function, tumor cell survival, and inflammatory responses of TAMs, all of which need to be proven in individual cell types. Moreover, if a single amino acid residue within this motif is found to mainly contribute to YKL-40's function, this could help screen new therapeutic agents aiming at this specific element. Finally, one of the most challenging research approaches is to identify the membrane receptor(s) specific for YKL-40 binding, which would not only provide new mechanistic insights into YKL-40's action, but also establish proof-of-principle for offering a novel mechanistically-directed target in treatment of a wide spectrum of cancers as well as other types of diseases. Therefore, gaining such important knowledge about pathological activities and molecular mechanisms of YKL-40 will unequivocally pave a fundamental way toward an advanced platform able to notably improve the current diagnosis, prognosis, and therapy of multiple human diseases that are associated with increased levels of YKL-40.

### Conflict of interest statement

The author declares that the research was conducted in the absence of any commercial or financial relationships that could be construed as a potential conflict of interest.

## Abbreviations

VEGF: vascular endothelial growth factor
Flk-1: VEGF receptor 2
PDGF: platelet-derived growth factor
EGF: epidermal growth factor
bFGF: basic fibroblastic growth factor
mAY: neutralizing anti-YKL-40 antibody
FAK: focal adhesion kinase
MAPK: mitogen-activated protein kinase
Erk: Extracellular signal-regulated kinase
PI3K: phosphoinositide 3-kinase
AKT: protein kinase B
JNK: c-Jun N-terminal kinase
TAM: tumor-associated microphage
GBM: glioblastoma
GSDC: glioblastoma stem-differentiated cells
HMVEC: human microvascular endothelial cells
VE-cad: vascular endothelial cadherin
N-cad: neural cadherin
ER: estrogen receptor
PR: progesterone receptor
Her2/*neu*: human epidermal growth factor receptor 2
MMP: metalloproteinase
shRNA: mall hairpin
HS: heparan sulfate.
